# Cs_2_NaGaBr_6_: a new lead-free and direct band gap halide double perovskite

**DOI:** 10.1039/d0ra01764g

**Published:** 2020-05-05

**Authors:** Yasir Saeed, Bin Amin, Haleema Khalil, Fida Rehman, Hazrat Ali, M. Imtiaz Khan, Asif Mahmood, M. Shafiq

**Affiliations:** Department of Physics, Abbottabad University of Science and Technology Havelian Abbottabad KPK Pakistan yasir.saeed@kaust.edu.sa +92 3454041865; Department of Physics, Hazara University Mansehra Pakistan; College of Engineering, Chemical Engineering Department, King Saud University P. O. Box 800 Riyadh 11421 Saudi Arabia

## Abstract

In this work, we have studied new double perovskite materials, A_2_^1+^B^2+^B^3+^X_6_^1−^, where A_2_^1+^ = Cs, B^2+^ = Li, Na, B^3+^ = Al, Ga, In, and X_6_^1−^. We used the all electron full-potential linearized augmented plane wave (FP-LAPW+lo) method within the framework of density functional theory. We used the mBJ approximation and WC-GGA as exchange–correlation functionals. We optimized the lattice constants with WC-GGA. Band structures were calculated with and without spin–orbit coupling (SOC). Further, band structures for Cs_2_LiGaBr_6_ and Cs_2_NaGaBr_6_ were calculated with SOC + mBJ to correct the band gap values with respect to experimental value. We obtained direct bandgaps at Γ-point of 1.966 eV for Cs_2_LiGaBr_6_ and 1.762 eV for Cs_2_NaGaBr_6_, which are similar to the parent organic–inorganic perovskite (MAPI) CH_3_NH_3_PbI_3_ (*E*_g_ = 1.6 eV). Total and partial density of states were analyzed to understand the orbital contribution of Cs, Na, Li, Ga and Br near the Fermi level. The optical properties in terms of real and imaginary *ε*, refractive index *n*, extinction coefficient *k*, optical conduction *σ*, absorption *I*, and reflectivity *R* were calculated. A study of the elastic and mechanical properties shows that both materials are thermodynamically stable. A stable, direct bandgap and a gap value close to those of MAPI make Cs_2_NaGaBr_6_ a great competitor in the Pb-free hybrid perovskite solar cells world.

## Abstract 2

In this work, we have studied new double perovskite materials, A_2_^1+^B^2+^B^3+^X_6_^1−^, where A_2_^1+^ = Cs, B^2+^ = Li, Na, B^3+^ = Al, Ga, In, and X_6_^1−^.

## Graphical abstract

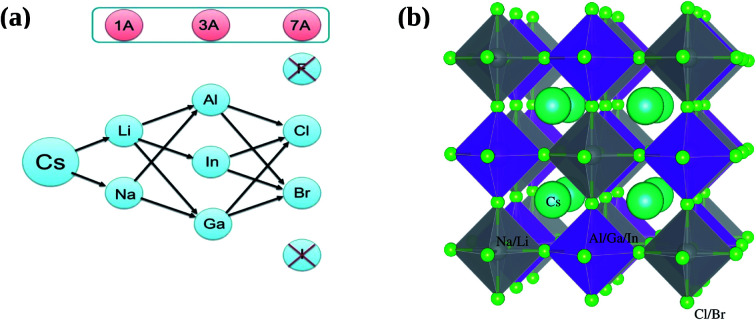

## 1. Introduction

The inevitable crisis in energy and climate change associated with conventional fossil fuel usage needs renewable energy technologies to be created. The organic–inorganic hybrid halide perovskites CH_3_NH_3_PbX_3_ (where X = I, Cl, Br) have attracted much attention in high-efficiency solar cell applications, solar fuel generation and solar hydrogen production.^1–3^ However, the poor stability and toxicity due to Pb (lead) have limited their commercialization on a large scale, which makes it necessary to search for Pb free and stable new materials as alternatives. Dual halides of perovskite have stimulated great attention.^3–15^

The double-perovskite halides are a large family of quaternary halides. The vast geometrical range of these materials poses opportunities to find new solar and optoelectronic materials; however, it also raises challenges in testing a large number of compounds to classify the most promising with ideal energetic, structural and electronic properties for solar cell applications. A series of double-perovskite halide compounds such as Cs_2_AgBiCl_6_ and Cs_2_AgBiBr_6_ have recently attracted considerable interest as promising alternatives to the CH_3_NH_3_PbI_3_ solar absorber material because they are Pb-free and can have improved stability.^16^ Dan and co-worker predicted thermodynamic stability of a number of double-perovskite halides using density functional theory (DFT).^17^

**Fig. 1:**
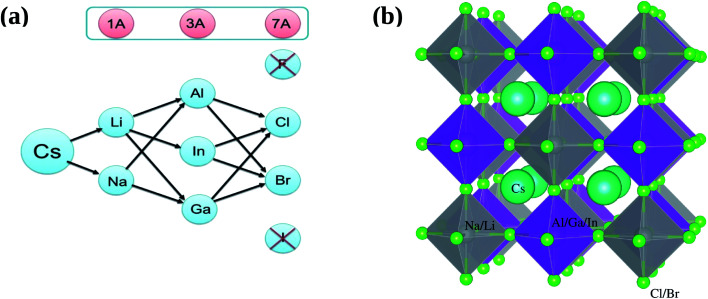
(a) Possible combination of A_2_^1+^B^2+^B^3+^X_6_^1−^ and (b) prospective view of double perovskites crystal structures A_2_^1+^B^2+^B^3+^X_6_^1−^, where A_2_^1+^ = Cs, B^2+^ = Li, Na, B^3+^ = Al, Ga, In, and X_6_^1−^ = Cl, Br.

All inorganic double perovskite material shows much attention to replace lead based perovskite material due to their three dimensionality and non toxic nature. Recently, Giustino and Snaith,^15^ predict new elements forming halide double perovskites. We are choosing one class of these materials from there proposed materials, see Fig. 1a, except F (due to large bandgap) and I (due to instability) as A_2_^1+^B^2+^B^3+^X_6_^1−^.

In this context, we investigate the stable and direct bandgap double perovskite material A_2_^1+^B^2+^B^3+^X_6_^1−^, where A_2_^1+^ = Cs, B^2+^ = Li, Na, B^3+^ = Al, Ga, In, and X_6_^1−^ = Cl, Br, see Fig. 1b. We will study structural as well as electronic properties of these materials and deeply analyzed optical and thermodynamic properties of selected materials which lies close to the bandgap of CH_3_NH_3_PbX_3_.

## 2. Computational details

Our computational calculations are based on density functional theory (DFT), using the full-potential linearized augmented plane wave plus local orbitals (FP-LAPW+lo) method as embedded in the WIEN2k scientific code.^18^ We employed more accurate exchange–correlation potential flavor as generalized gradient approximation (WC-GGA) for the calculation of the structural, electronic and thermodynamic properties.^19^ In addition, for obtaining accurate bandgap, we also applied the modified Becke–Johnson (mBJ)^20,21^ schemes over the WC-GGA. In the past, the mBJ scheme yields promising electronic band structures and bandgap values for lead halide perovskites and various types of semiconductors compare to experimental values.^22–32^ In this paper we demonstrate that mBJ over WC-GGA give results close to experimental one for Cs_2_AgBiCl_6_ and than applied to predicted A_2_^1+^B^2+^B^3+^X_6_^1−^. Electronic calculations have been performed with *k*-mesh of 13 × 13 × 13 and finer *k*-mesh of 24 × 24 × 24 for calculating the optical properties. All structures were optimized with energy convergence tolerance of 10^−5^ Ryd.

## 3. Results and discussion

### 3.1 Structural properties

According to proposed plan of study the prospective view of crystal structure of possible double perovskites A_2_^1+^B^2+^B^3+^X_6_^1−^ where A_2_^1+^ = Cs, B^2+^ = Li, Na, B^3+^ = Al, Ga, In and X_6_^1−^ = Cl, Br, is shown in Fig. 1b. For simplicity, we categorized all 12 possible compounds into two categories, one is Li-based and second is Na-based, as shown in Fig. 1a. First of all, structural parameters like lattice constant and bulk modulus are obtained by minimizing the total energy *versus* volume for A_2_^1+^B^2+^B^3+^X_6_^1−^ compounds. We use experimental lattice parameter of Cs_2_AgBi(Br/Cl)_6_ (ref. 16) as our starting point. The Birch–Murnaghan's equation of states^33^ is used for optimization of structural parameters of these compounds are listed in Table 1.

**Table 1:** Optimized structural parameters and bandgap values of studied double perovskites A_2_^1+^B^2+^B^3+^X_6_^1−^

	Optimized lattice constant (Å)	Volume (bohr^3^)	Bulk modulus (*B*) (GPa)	Band gap (eV)
Cs_2_LiAlCl_6_	9.80	1588.95	39.58	3.222
Cs_2_LiGaCl_6_	9.87	1621.55	36.73	1.87
Cs_2_LiInCl_6_	10.10	1790.25	35.15	2.655
Cs_2_LiAlBr_6_	10.41	1901.34	31.53	1.889
Cs_2_LiGaBr_6_	10.7867	2117.34	27.10	0.731
				1.966 (with mBJ + SOC)
Cs_2_LiInBr_6_	10.7947	2122.13	20.45	1.502
Cs_2_NaAlCl_6_	9.99	1685.68	36.24	3.039
Cs_2_NaGaCl_6_	10.09	1729.68	33.66	1.691
Cs_2_NaInCl_6_	10.37	1882.69	32.12	2.491
Cs_2_NaAlBr_6_	10.66	2045.57	25.25	1.728
Cs_2_NaGaBr_6_	10.96	2221.04	22.80	0.451
				1.762 (with mBJ + SOC)
Cs_2_NaInBr_6_	10.97	2229.17	20.64	1.401

Optimized lattice constants for all studied compounds are between 9.8 Å to 10.97 Å which are quite close to experimentally synthesized double perovskites like Cs_2_AgBiCl_6_ (10.77 Å) and Cs_2_AgBiB_6_ (11.27 Å).^16^

The optimized bulk modulus (*B*) for Cs_2_LiAlCl_6_ is 39.58 GPa which is highest among all other compounds. In contrast, Cs_2_LiInBr_6_ has smallest value of *B* is 20.45 GPa which shows the hardness of these double perovskites. There is no theoretical or experimental data available for comparison of these optimized compounds.

After optimizing the lattice constants, it is important to characterize these compounds on the basis of band gap values, which are close to the best hybrid organic–inorganic perovskites.

### 3.2 Electronic band structure

To study about the electronic behavior of materials, band structure plays very important role. The electronic nature of materials can be disclosed using band structure such as metallic or semiconducting *etc.* Physical properties of solids such as electrical resistivity and optical behavior, can be explain successfully from electronic band structure and forms a foundation for solid state devices (transistors, solar cells, *etc.*). Therefore, band structure is calculated to realize electronic behavior of all Cs_2_LiB^3+^X_6_^1−^ and Cs_2_NaB^3+^X_6_^1−^ compounds where B^3+^ = Al, Ga, In and X_6_^1−^ = Cl, Br. We can calculate band gap for all studied compounds with three different ways. First, band gap obtained by simple scf calculated where we did not incorporate spin–orbit coupling (SOC). Second one is to incorporate spin–orbit coupling to find the band gap values close to experimental values. Also we find splitting of band near the valence band maxima (VBM) or conduction band minima (CBM). Third, even if we could not match our bandgap values with experimental, we use different method to correct the band gap. Here we use mBJ method, which is quite expensive calculation with respect to time taken by CPU's in computational.

For our compounds, the calculated band gap of all materials without SOC and mBJ + SOC are given in Table 1. Clearly we can see, that only Cs_2_LiGaBr_6_ and Cs_2_NaGaBr_6_ have small band gap, 0.731 eV and 0.451 eV respectively. While all other compounds have band gap above 1.4 eV without SOC and mBJ + SOC. We observe in double perovskite materials Cs_2_AgBiCl_6_ and Cs_2_AgBiBr_6_ (as a test calculation) have band gap 2.77 eV and 2.19 eV respectively.^16^ Which we reproduced by WIEN2k and for Cs_2_AgBiCl_6_ case, we find band gap values for without SOC and with SOC, mBJ and mBJ + SOC, and values are 1.702 eV, 1.629 eV, 3.088 eV and 2.66 eV respectively. Also for Cs_2_AgBiBr_6_, without SOC (1.16 eV), with SOC (1.163 eV), mBJ (2.165 eV) and mBJ + SOC (1.947 eV). These results clearly shows that in double perovskites, WIEN2k underestimate the band gap values substantially until we incorporate mBJ + SOC. So our target is to find new Pb free material, whose band gap is close to 1.6 eV to replace CH_3_NH_3_PbI_3_ at room temperature.^15^

We concluded by analyzing Table 1, that if we apply mBJ + SOC on material whose band gap is already above 1.4 eV, results in larger band gap value probably >2 eV. So we decided two candidates remaining, Cs_2_LiGaBr_6_ (0.731 eV) and Cs_2_NaGaBr_6_ (0.451 eV).

**Fig. 2:**
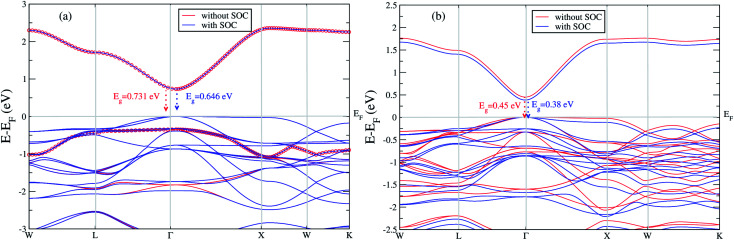
The calculated band structure of (a) Cs_2_LiGaBr_6_ and (b) Cs_2_NaGaBr_6_ with and without spin–orbit coupling.

We extend our further study only focusing these two materials. We calculated band gap for Cs_2_LiGaBr_6_ with and without SOC. The band structures are given in Fig. 2a. The band gap values for Cs_2_LiGaBr_6_ with and without SOC are 0.731 eV (red color) and 0.646 eV (blue color) respectively. Which indicate the importance of SOC in Cs_2_LiGaBr_6_, SOC slightly decrease in band gap value (see Fig. 2a), we can see that spin–orbit coupling splitting VBM in majority, while a minor splitting around 2 eV in CBM.

**Fig. 3:**
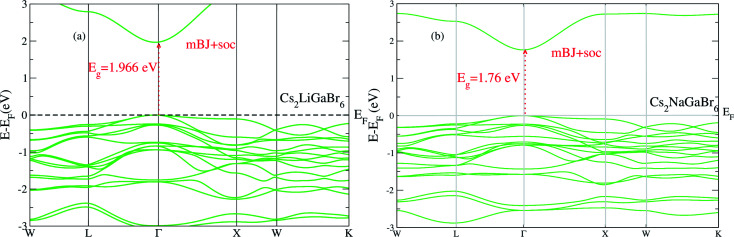
The calculated band structure of (a) Cs_2_LiGaBr_6_ and (b) Cs_2_NaGaBr_6_ with modified band gap using mBJ + SOC scheme.

On the other hand in case of Cs_2_NaGaBr_6_, the band gap value, without spin–orbit coupling (0.45 eV) and decreasing with spin–orbit coupling (0.38 eV), (see Fig. 2b). Also if we examine the Fig. 2b more closely, we can see that VBM is splitting near Fermi energy at Γ point as well as SOC shifting CBM towards Fermi level. However, the SOC effect is spread over whole band structure in VBM too. Next we calculated the band structure of Cs_2_LiGaBr_6_ and Cs_2_NaGaBr_6_ with mBJ + SOC, shown in Fig. 3a and b. The calculated direct band gap values for Cs_2_LiGaBr_6_ and Cs_2_NaGaBr_6_ are 1.966 eV and 1.76 eV, respectively.

Among these two final materials Cs_2_NaGaBr_6_ and Cs_2_LiGaBr_6_ band gap values are more close to the experimental value of CH_3_NH_3_PbI_3_ (Cl, Br). As we know that a band gap between 1.0 and 1.7 eV, electrons can be released without creating too much heat and makes an effective solar semiconductor in this range. The photon energy of light varies according to the different wavelengths of light. However, the nature of band gaps of these compounds were indirect. While recently studied double perovskites have direct band gap but band gap values are beyond this range and some have band gap values in effective range but nature of band gap were indirect, which is not ideal for applications in solar cell.^9^

A high carrier mobility is strongly desirable for competent electronic and optoelectronic devices.^34^ The effective masses of MAPI are <0.2 *m*_e_ and the carrier mobility of <100 cm^2^ V^−1^ s^−1^ is moderate in comparison to conventional semiconductors such as Si or GaAs (>1000 cm^2^ V^−1^ s^−1^).^35,36^ The balanced effective mass in MAPI may lead to ambipolar conductivity in perovskite solar cells, which facilitates the p–i–n junction solar cell.^37^ The conduction and valence band fitting to parabola using Fig. 3, provides the effective mass (m*) of Cs_2_Li(Na)GaBr_6_. Here we used the deformation potential theory for calculating the effective masses for holes 
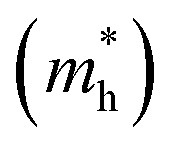
 and electrons 
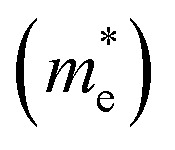
. The calculated effective masses of Cs_2_Li(Na)GaBr_6_ are 0.154 (0.176) and 0.176 (0.186) for 
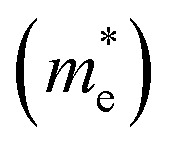
 and 
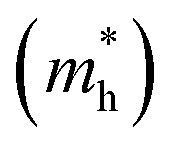
, respectively. From the calculated effective masses its cleared that 
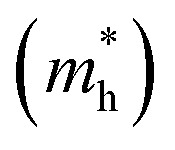
 is slightly greater than that of 
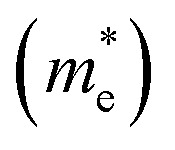
 but overall <0.2 *m*_e_, which is in good agreement with MAPI and keeping ambipolar conductivity due to balanced effective mass in Cs_2_NaGaBr_6_. A greater transport of charge carriers is followed by owing the smaller effective mass.

### 3.3 Density of states (DOS)

**Fig. 4:**
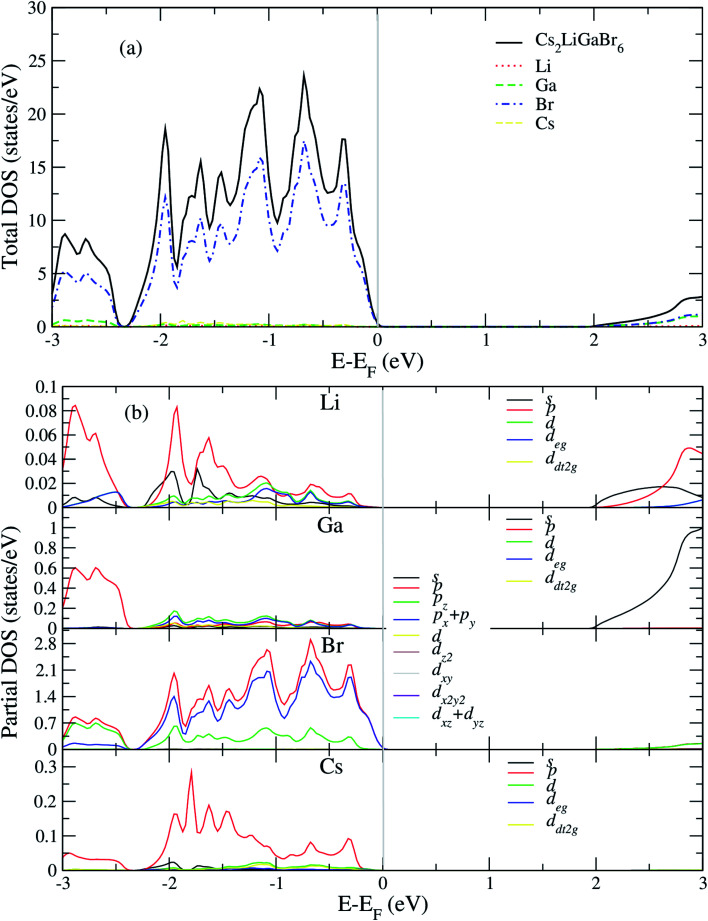
Calculated (a) total and (b) partial DOS of Cs_2_LiGaBr_6_.

**Fig. 5:**
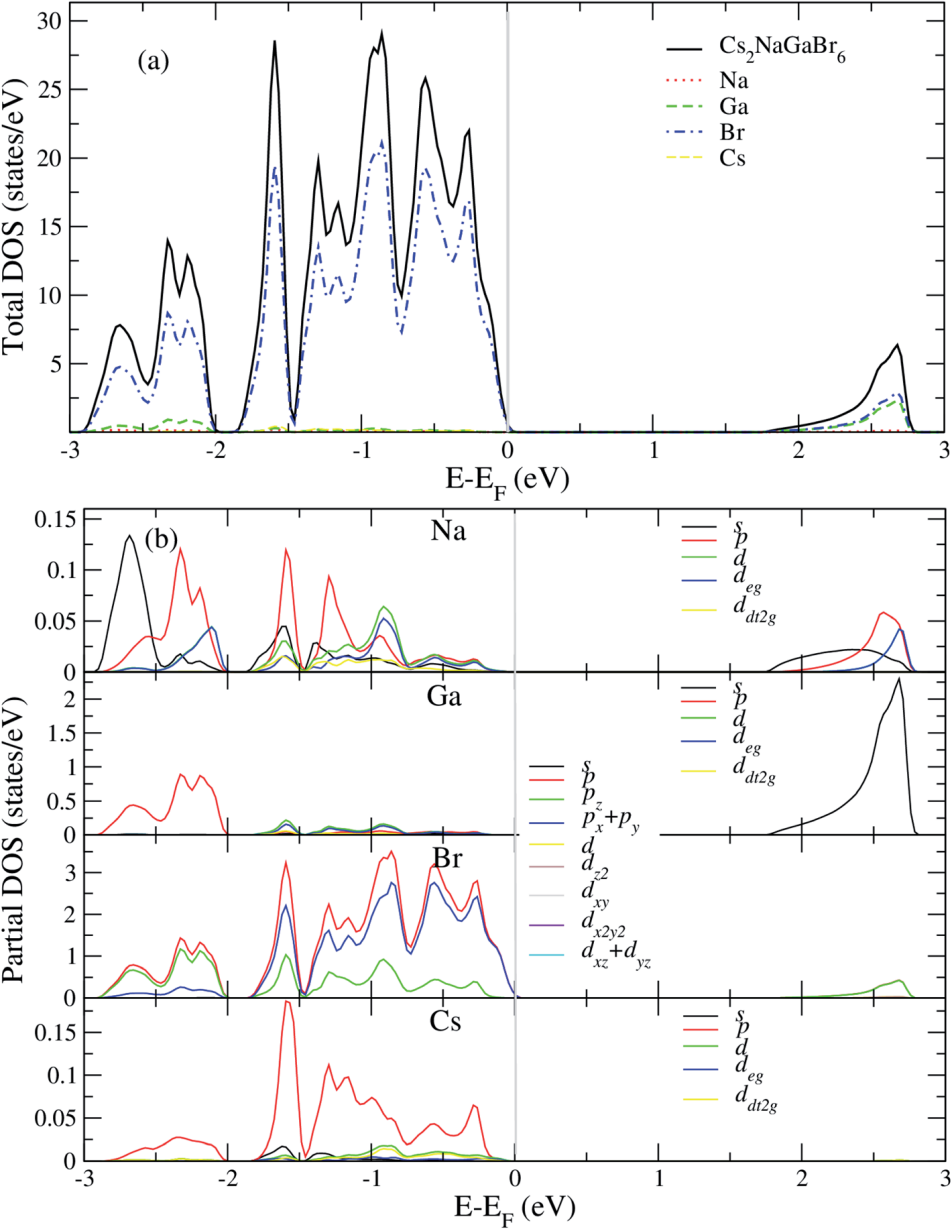
Calculated (a) total and (b) partial DOS of Cs_2_NaGaBr_6_.

After analyzing band structure and band gap values, now we are moving into density of states of these materials to find contribution of elements (Cs, Na, Ga, Br) into CBM and VBM Fig. 4 and 5 shows the total and partial DOS of Cs_2_LiGaBr_6_ and Cs_2_NaGaBr_6_ respectively. Fig. 4a clearly shows that the valence band maxima completely contributed in Br atom, while a conduction band minimum is jointly shared in Br and Ga atoms. Similarly, in case of Cs_2_NaGaBr_6_ while Li and Na atoms in both compounds are mainly contributed in band gap value, see Fig. 5a.

To know, which Br or Ga orbital play role in valence band maxima and conduction band maxima, we further calculate partial DOS, as shown in Fig. 4b (Cs_2_LiGaBr_6_) and Fig. 5b (Cs_2_NaGaBr_6_). From Fig. 4b, we can clearly see that Li-p orbital is dominant as compared to s and d-orbitals of Li in VBM and CBM, while its magnitude is very small as compared to all other atoms. Also Ga-s orbital shows its significant CBM, while Ga-p and d orbitals are mainly but tiny contributed in VBM. For Br, if p-orbital is dominated all the valence band maxima up to 2 eV, then Br-p_*x*_ + p_*y*_ orbital play key role in majority peaks in valence band maxima. However, Br-d orbital does not have any contribution in VBM and CBM due to lying deep in valence band. Cs-p orbital also contributing in constructing VBM. Its contribution is larger than Li and Ga atoms. Similarly, behavior observed in partial DOS of Cs_2_NaGaBr_6_, only one major difference is that in Cs_2_NaGaBr_6_ conduction band minima are completely dominated by Ga-s orbitals and VBM is dominated by Br-p orbital, see Fig. 5b.

### 3.4 Optical properties

The linear optical properties are described using the frequency-dependent dielectric *ε*(*ω*) = *ε*_1_ + i*ε*_2_(*ω*) function. The equation of frequency-dependent imaginary part of dielectric function *ε*_2_(*ω*) for cubic crystal is provided as:^38^
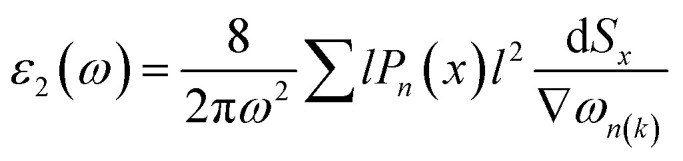
where *P*(*x*) is the dipole momentum matrix of the exterior band transformation. The joint DOS and transition momentum matrix components heavily correlate the dielectric feature *ε*_2_(*ω*). The real part of the dielectric function *ε*_1_(*ω*) can be acquired from the *ε*_2_(*ω*) relationship provided in ref. 39 by Kramers–Kronig.
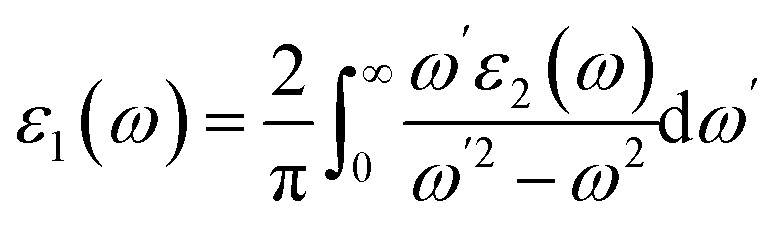


**Fig. 6:**
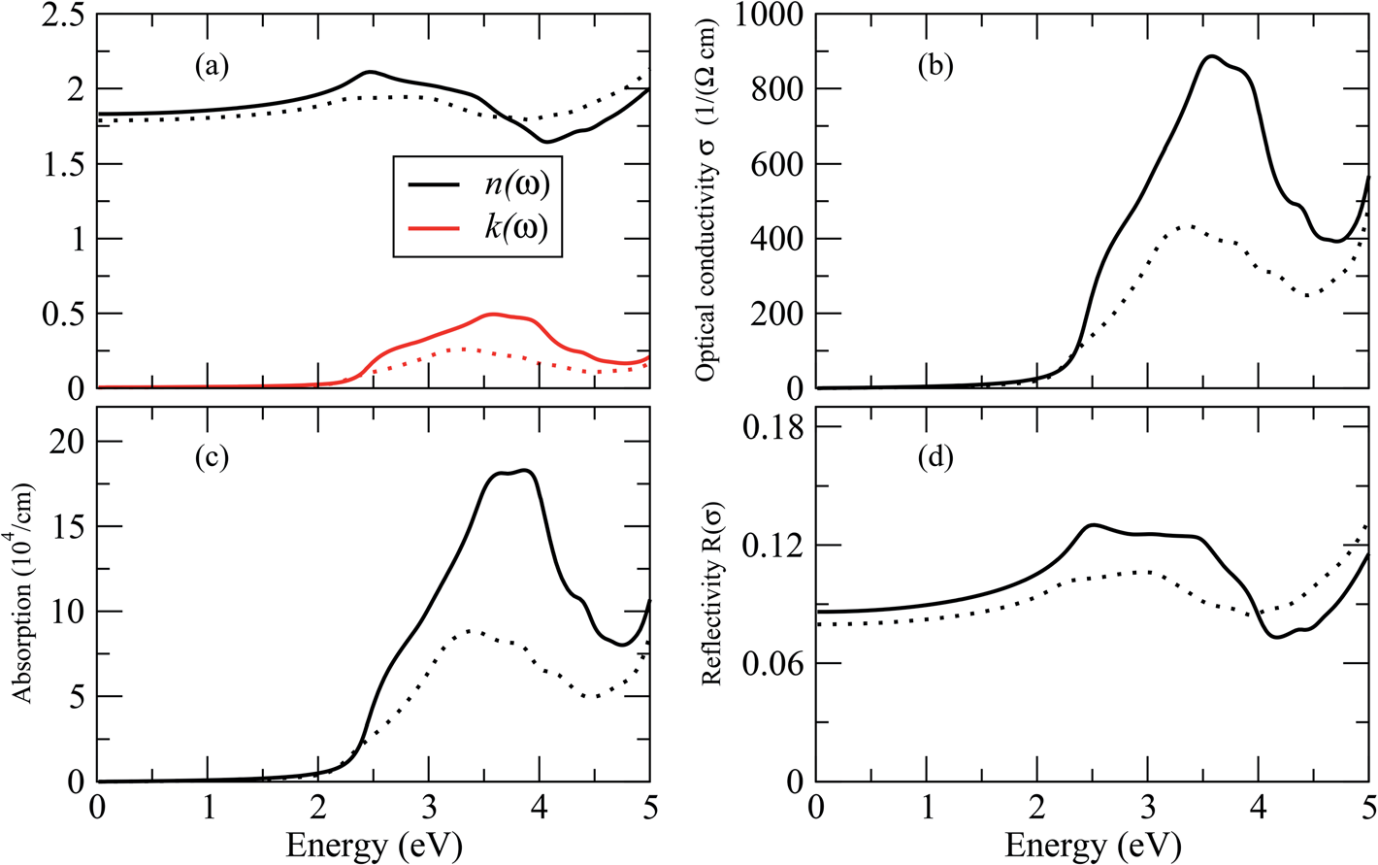
Calculated optical properties of Cs_2_LiGaBr_6_ (dotted line) and Cs_2_NaGaBr_6_ (solid lines). (a) Optical constant (*n*, *k*), (b) optical conductivity *σ*, (c) absorption coefficient *I* and (d) reflectivity *R*.

By dispersing real and imaginary components of dielectric features, other optical parameters such as refractive indices, reflectivity, optical conductivity, absorption coefficient and so on can be calculated. Cs_2_LiGaBr_6_ and Cs_2_NaGaBr_6_'s linear optical characteristics were investigated by calculating the optical parameters, dielectric constant *ε*_0_(*ω*), refractive index (*n*), extinction coefficient (*k*), absorption coefficient *I*(*ω*), reflectivity *R*(*ω*), and optical conductivity *σ*(*ω*) shown in Fig. 6. The static dielectric constant *ε*_1_(0) is 1.6 eV for Cs_2_LiGaBr_6_ and 1.7 eV for Cs_2_NaGaBr_6_ without any contribution from the lattice vibration.

The complex refractive index as a function of energy in eV is shown in Fig. 6. The real part of refractive index *n* is a measure of phase velocity in a medium of electromagnetic (EM) wave and imaginary part *k* control the effect of EM wave traveling through a material. The calculated static refractive index *n*(0) are around ∼1.8 for both materials while the highest values of *n*(*ω*) and *k*(*ω*) for Cs_2_LiGaBr_6_ are 3.0 eV and 3.25 eV respectively. In case of Cs_2_NaGaBr_6_ these values are 2.5 eV and 3.5 eV for *n*(*ω*) and *k*(*ω*). The optical conductivity corresponds to the conduction of electrons produced when a photon of a certain frequency is incident upon a material. Fig. 6 show maximum of optical conductivity (*σ*) of 616 [Ω cm]^−1^ is about at 8.6 eV for Cs_2_LiGaBr_6_ and first peak of 417 [Ω cm]^−1^ at 3.4 eV. While for Cs_2_NaGaBr_6_ first peak of 915 [Ω cm]^−1^ at 3.6 eV and maximum (*σ*) of 6015 [Ω cm]^−1^ at 9.2 eV. Cs_2_NaGaBr_6_ has high optical conductivity in the visible light region (1.65–3.1 eV), which is important for solar cell materials. The solar energy conversion efficiency determines from absorption coefficient and it indicates how far light of a specific frequency can penetrate into the material before absorption. The absorption coefficient spectrum *I*(*ω*), started at 1.96 eV and its maximum arises at 3.26 eV (380.32 nm) for Cs_2_LiGaBr_6_ and for Cs_2_NaGaBr_6_ its started from 1.76 eV and it maxima arises at 3.72 eV (333.29 nm). The absorption coefficient values rapidly increases when the incident photon energy reaches the absorption edge. But at the other side, in the high-energy zone, the absorption coefficients are rapidly decreasing and this is a typical feature of semiconductors. In visible region Cs_2_NaGaBr_6_ has more absorption that Li-based Cs_2_LiGaBr_6_. While maximum of reflectivity *R*(*ω*) is at about 2.89 eV for Cs_2_LiGaBr_6_ and for Cs_2_NaGaBr_6_ (0.13 or 13%) at 2.54 eV.

### 3.5 Elastic and mechanical properties

The effective elastic constants provide an appropriate description of materials for desirable practical applications. The elastic constants describe the structure stability and response of the materials to external forces.

The calculated elastic constants for Cs_2_LiGaBr_6_ and Cs_2_NaGaBr_6_ are presented in Table 2. No experimental results are available for comparison to our predicted values. The Born^40^ stability criteria for cubic crystals *i.e.*, *C*_11_ + 2*C*_12_ > 0, *C*_11_ − *C*_12_ > 0, *C*_11_ > 0, *C*_44_ > 0 and *C*_12_ < *B* < *C*_11_ are fulfilled by these materials. This justified that these materials are elastically stable against deforming force.

**Table 2:** The calculated values of elastic constants *C*_11_, *C*_12_ and *C*_44_, Voigt's shear modulus *G*_V_, Reuss's shear modulus *G*_R_, Hill's shear modulus *G*_H_, *B*/*G* ratio, Cauchy pressure (*C*′′), Poisson's ratio (*v*), anisotropy constant (*A*), and shear constant (*C*′)

Comp.	Cs_2_LiGaBr_6_	Cs_2_NaGaBr_6_
*C*_11_	46.42	38.97
*C*_12_	19.48	14.16
*C*_44_	18.82	14.52
*G*_V_	17.20	13.68
*G*_R_	16.72	13.60
*G*_H_	16.96	13.64
*Y*	42.85	34.10
*B*/*G*	1.65	1.65
*C*′′	−0.62	−0.36
*v*	0.25	0.25
*A*	1.41	1.17
*C*′	16.59	12.41

Further, elastic constants are used to calculate the important mechanical properties of materials under study using standard relations^41,42^ and presented in Table 2.

The shear modulus *G*_H_ is the arithmetic mean of the *G*_R_ and *G*_H_ shear moduli measure the plastic deformation of the material to applied stress. The higher value of *G*_H_ and Young modulus *Y* of Cs_2_LiGaBr_6_ indicate that this material offer more resistance to plastic deformation and hence stiffer than Cs_2_NaGaBr_6_. Pugh^43^ (*B*/*G* ratio) reflect the brittle and ductile behavior of materials. It is clear from the Table 2 that *B*/*G* ratio for both materials is less than critical value of 1.75, hence both materials show brittle nature. The negative/positive value of Cauchy's pressure (*C*′′ = *C*_12_ − *C*_44_) also indicate the brittle/ductile behavior of materials. The negative value of Cauchy's pressure (Table 2) further confirm the brittle behavior of Cs_2_LiGaBr_6_ and Cs_2_NaGaBr_6_. Poisson's ratio *v* measures the stability of a crystal against compressibility. The smaller value of Poisson's ratio indicate that these materials is relatively stable against shear stress. The calculated values of anisotropic factor for Cs_2_LiGaBr_6_ and Cs_2_NaGaBr_6_ are given in Table 2. The deviation of *A* from unity measure the degree of anisotropy of material. It is evident from the table that anisotropic factor is greater than unity, hence their properties vary in different crystallographic directions. Shear constant or tetragonal shear modulus *C*′ define the dynamical stability of materials. It also describes the stability to tetragonal distortion. For dynamical stability *C*′ > 0.^44^ The positive value of *C*′ for the materials under investigation (Table 2) predicted that these materials are mechanically stable.

## 4. Conclusion

In this study, double perovskites A_2_^1+^B^2+^B^3+^X_6_^1−^, where A_2_^1+^ = Cs, B^2+^ = Li,Na, B^3+^ = Al, Ga, In have been investigated using all-electron full potential linearized augmented plane wave (FP-LAPW+lo) method within the frame work of density functional theory. We optimize all materials using WC-GGA approximation along with band gaps. In addition to this, we employed modified Becke–Johnson band gap correction in order to obtain correct band gap values with respect to experimental material (CH_3_NH_3_PbI_3_). After carefully optimizing, the volume optimization and predicted lattice constants of A_2_^1+^B^2+^B^3+^X_6_^1−^ were obtained. We calculated band gap of these materials. Among, these materials only Cs_2_LiGaBr_6_ and Cs_2_NaGaBr_6_ have band gap values 0.731 eV and 0.45 eV, respectively. Rest of materials have band gap above 1.4 eV, which may further increase over 2 eV on applying mBJ correction. We also study the effect of spin–orbit coupling (SOC) in these materials. We found that SOC effect is only lowering the bands in energies. Due to spin–orbit coupling, band gap values are reduced from 0.731 to 0.646 eV for Cs_2_LiGaBr_6_ and from 0.45 to 0.38 eV for Cs_2_NaGaBr_6_. Total density of state shows that Br is major contributor in valence band maxima and Ga contributes in conduction band minima. While in partial density of state (PDOS) reveal that Br-p orbital bands are contributing while Ga s-orbital. We have calculated optical spectra to study absorption for Cs_2_NaGaBr_6_ has more absorption coefficient than Cs_2_LiGaBr_6_. So we concluded that Cs_2_NaGaBr_6_ is more suitable candidate among studied double perovskite compounds on the basis of band gap value and optical absorption for solar cell application. Stable, direct band gap and value of gap close to MAPI makes Cs_2_NaGaBr_6_ a great competitor in Pb-free hybrid perovskites solar cell world.

## Conflicts of interest

There are no conflicts to declare.

## Supplementary Material
