# Correction: Cs_2_NaGaBr_6_: a new lead-free and direct band gap halide double perovskite

**DOI:** 10.1039/d0ra90062a

**Published:** 2020-05-27

**Authors:** Yasir Saeed, Bin Amin, Haleema Khalil, Fida Rehman, Hazrat Ali, M. Imtiaz Khan, Asif Mahmood, M. Shafiq

**Affiliations:** Department of Physics, Abbottabad University of Science and Technology Havelian Abbottabad KPK Pakistan yasir.saeed@kaust.edu.sa +92 3454041865; Department of Physics, Hazara University Mansehra Pakistan; College of Engineering, Chemical Engineering Department, King Saud University P. O. Box 800 Riyadh 11421 Saudi Arabia

## Abstract

Correction for ‘Cs_2_NaGaBr_6_: a new lead-free and direct band gap halide double perovskite’ by Yasir Saeed *et al.*, *RSC Adv.*, 2020, **10**, 17444–17451, DOI: 10.1039/D0RA01764G.

The author regrets that the funding information was incorrectly shown in the acknowledgements section of the original manuscript. The corrected funding acknowledgement is as shown below.

The authors would like to thank IT Department of National Center for Physics (NCP), Islamabad for supplying computational resources. The author Asif Mahmood extends their appreciation to the Deanship of Scientific Research at King Saud University for funding the work through the research group project No. RGP-311.

The Royal Society of Chemistry apologises for these errors and any consequent inconvenience to authors and readers.

## Supplementary Material

